# Xylosyltransferase-I Regulates Glycosaminoglycan Synthesis during the Pathogenic Process of Human Osteoarthritis

**DOI:** 10.1371/journal.pone.0034020

**Published:** 2012-03-30

**Authors:** Narayanan Venkatesan, Lydia Barré, Mustapha Bourhim, Jacques Magdalou, Didier Mainard, Patrick Netter, Sylvie Fournel-Gigleux, Mohamed Ouzzine

**Affiliations:** UMR 7561 CNRS-Université Nancy 1, Faculté de Médecine, Vandœuvre-lès-Nancy, France; King's College London, United Kingdom

## Abstract

Loss of glycosaminoglycan (GAG) chains of proteoglycans (PGs) is an early event of osteoarthritis (OA) resulting in cartilage degradation that has been previously demonstrated in both huma and experimental OA models. However, the mechanism of GAG loss and the role of xylosyltransferase-I (XT-I) that initiates GAG biosynthesis onto PG molecules in the pathogenic process of human OA are unknown. In this study, we have characterized XT-I expression and activity together with GAG synthesis in human OA cartilage obtained from different regions of the same joint, defined as “normal”, “late-stage” or adjacent to “late-stage”. The results showed that GAG synthesis and content increased in cartilage from areas flanking OA lesions compared to cartilage from macroscopically “normal” unaffected regions, while decreased in “late-stage” OA cartilage lesions. This increase in anabolic state was associated with a marked upregulation of XT-I expression and activity in cartilage “next to lesion” while a decrease in the “late-stage” OA cartilage. Importantly, XT-I inhibition by shRNA or forced-expression with a pCMV-XT-I construct correlated with the modulation of GAG anabolism in human cartilage explants. The observation that XT-I gene expression was down-regulated by IL-1β and up-regulated by TGF-β1 indicates that these cytokines may play a role in regulating GAG content in human OA. Noteworthy, expression of IL-1β receptor (IL-1R1) was down-regulated whereas that of TGF-β1 was up-regulated in early OA cartilage. Theses observations may account for upregulation of XT-I and sustained GAG synthesis prior to the development of cartilage lesions during the pathogenic process of OA.

## Introduction

Osteoarthritis (OA) is a chronic, progressive, degenerative disorder of joint characterized by breakdown of articular cartilage leading to functional failure of the joint. OA is characterized by progressive loss of extracellular matrix (ECM) proteins, causing mechanical disruption of the articular cartilage surface, together with subchondral bone sclerosis and osteophyte formation [1]. One of the earliest changes in OA development is the loss of matrix proteoglycans (PGs) [2], and studies have monitored loss of aggrecan, the predominant cartilage PG, which provides cartilage the characteristic mechanical properties of compressibility and resilience [3]. Evidence from studies in vitro and in vivo indicates that interleukin-1β (IL-1β) and tumor necrosis factor-α (TNF-α) are the predominant cytokines involved in the initiation and progression of articular cartilage destruction [4], [5]. IL-1β, besides its catabolic activity, inhibits PG synthesis in cartilage, a process observed in early stages of OA [4]–[6]. Notably, loss of PG anabolism not only participates in alteration of the mechanical properties of cartilage but may also impair the chondrocyte metabolism and ability to repair the damage. Indeed, PGs control diverse cellular and biological activities, including cell proliferation, migration and differentiation. Numerous studies suggest that most of the biological activity of PGs is mediated by their glycosaminoglycan (GAG) chains. In this context, GAG chains play important roles in growth factor signaling, cellular differentiation, morphogenesis, and pathophysiology [7], [8]. Therefore, alteration in the capacity of OA chondrocytes to restore the integrity of the cartilage matrix may be due to dysfunction of the regulatory pathway leading to formation of glycosaminoglycan chains.

In contrast to catabolism of OA cartilage, the synthesis side of OA has not received much attention. This is important because the repair potential of cartilage is gaining more attention as target for OA treatment. The initial event in response to injury by chondrocytes is an attempt to repair the damaged matrix by up-regulation of matrix synthesis including PGs. Proteomics studies have demonstrated elevated anabolism in OA cartilage associated with enhanced gene expression and biosynthesis of *COL2A*1 [9]. However, studies in animal models [10] and analysis of cartilage samples from OA patients [11], [12] indicated that this differential expression may depend upon the zone of cartilage analyzed in conjunction with the stage of OA progression. In a previous study [13] we showed that during the early phases of cartilage repair, following degradation by intraarticular injection of papaïn in rat knee, PG synthesis was increased concomitantly to up-regulation of gene expression of the xylosyltransfersase I (XT-I) responsible for the initiation of biosynthesis of the chondroitin sulfate and heparan sulfate GAG chains of PGs. shRNA inhibition of XT-I expression during recovery phases prevent cartilage repair indicating that XT-I play a central role in cartilage repair process [13]. Human studies relative to this issue, however, are lacking at present, and such information is essential in furthering our understanding of cartilage degeneration as well as to stimulate cartilage repair in OA patients. In this study, we examined the role of XT-I in OA disease. We found that XT-I regulates GAG synthesis in human cartilage during early onset and late stage of OA disease and that expression of XT-I gene is regulated by the proinflammatory cytokine IL-1β and by the cartilage anabolic factor TGF-β1. Interestingly, forced expression of this enzyme by gene transfer in late stage OA cartilage enhanced GAG synthesis and stimulate cartilage repair. Collectively, our results provide important insights into regulatory factors driving the changes in GAG synthesis that occur with the onset and progression of cartilage degeneration.

## Materials and Methods

### Human osteoarthritis cartilage samples, histology and scoring disease severity

Articular cartilage samples (femoral condyles and tibial plateau) were obtained from eight patients with OA (mean age 65±9 yr) undergoing total knee joint replacement. All OA patients were examined by a certified rheumatologist and were diagnosed as having OA, using the criteria of the American College of Rheumatology for OA [14]. This study was approved by our local Research Institution review board (Comission de la Recherche Clinique; registration number UF 9757 – CPRC 2004 – Cellules souches et chondrogénèse). The protocol conforms to the ethical guidelines of the Declaration of Helsinki and written informed consent has been obtained from each patient. Cartilage specimens (within 1 h of operation) were dissected using sterile techniques, and full-thickness cartilage was removed from all of the femoral condyle using a 6 mm biopsy punch. Extreme care was taken to avoid subchondral bone, osteophytic cartilage and fibrocartilage.

Cartilage samples were fixed for 24 h in 4% paraformaldehyde at 4°C, rinsed in running tap water for 1 minute, decalcified with RDO (Apex Engineering Products Corporation), in a volume of decalcifier equal to at least 10-times the tissue volume for 2 h. Samples were checked every 30 min and decalcified tissue was rinsed in running tap water for 5 min and further fixed in 4% paraformaldehyde. Samples were embedded in paraffin, and tissue sections (5 µm thick) were cut. Histological sections were rehydrated in a graded series of ethanol and stained with toluidine blue. Cartilage sections were graded histologically in line with Mankin's histological grading system for OA cartilages [15] to assess disease severity and cartilage damage based on surface fibrillation, matrix depletion and cellularity, and the integrity of the tidemark. No tidemarks were present in our cartilage samples.

### Construction of XT-I expression vector, transfection of chondrocyte and GAG synthesis

A human XT-I cDNA has been cloned and ligated into the unique *Hin*dIII-*Not*I sites of mammalian expression vector pCMV (Clontech, Mountain View, CA, USA) to generate pCMV-XT-I. Primary chondrocytes were isolated by sequential enzymatic digestion with pronase and collagenase of human femoral condyle cartilage [16] and maintained in DMEM-F12 mix medium in 6-well plates at 37°C in a humidified atmosphere supplemented with 5% CO_2_. Cells were then transfected with 1 µg of pCMV-XT-I expressing vector or pCMV empty vector at 80% confluence using Exgen 500 (Euromedex, Souffelweyersheim, France). At 48 h posttransfection, cells were labeled with 10 µCi/ml ^35^S-sulfate for the last 6 h of experimental period and PGs were extracted from cell layer and culture medium [17] with 4 M guanidine hydrochloride, precipitated using cetylpyridinium chloride, and sulfated GAG synthesis rate was measured by liquid scintillation counting [18].

### Real-time Quantitative PCR

All reagents and instruments used for RNA extraction were RNase-free. Total RNA from human cartilage samples was extracted by homogenization in Trizol solution and purified based on the manufacturer's instruction for the RNAeasy Kit (Qiagen, Cologne, Germany). Briefly, sterile scalpel was used for slicing of cartilage samples on ice to very small pieces (1 mm pieces) before homogenization process. Homogenization was performed in a 1.5 ml microcentrifuge tube on ice using a power homogenizer (Yellowline, DI25) with homogenization time of 10 s intervals for up to 1 min. The ratio of cartilage to Trizol used was 2% (w/v). In the case of cultured chondrocytes total RNA was prepared using RNAeasy kit. cDNA was synthesized from 0.2–1 µg of total RNA using oligodT primer and Power Script Reverse Transcriptase (Clontech). Quantitative PCR was performed using LightCycler system (Roche, Mannheim, Germany) and SYBR green detector (QIAGEN GmbH, Hilden, Germany). Transcripts were normalized to ribosomal protein S29. RT^2^ PCR primer sets for human XT-I, Aggrecan, IL-1R1, TGF-β1 and ribosomal protein S29 were from SuperArray (Frederick, MD, USA). PCR cycling parameters were 15 min at 95°C; 40 cycles of 10 s at 95°C, 35 s at 65°C, and 15 s at 72°C. Expression levels of target genes were normalized to ribosomal protein S29 RNA level.

### XT-I shRNA Transfection

Four shRNA vectors for XT-I and control were obtained from (SuperArray). The shRNA vectors were generated by ligating the hairpin oligonucleotides of the target sequences A: CCGGAGGTTTGTAGAATATGT, B: CGGGACAATGCAAGGTTCATT, C: GAGAGGCTATTCCGCAACTTT and D: TACCTTCTTTGCCCGCAAGTT, into SureSilencing™ shRNA plasmid. Each vector contains the shRNA under the control of the U1 promoter. The two vectors expressing shRNA of the sequences B and D were selected for their ability to significantly inhibit XT-I gene expression in primary chondrocytes. Full thickness cartilage (6 mm-biopsy punch) cultured in DMEM-F12 medium in 6-well plates at 37°C were transfected with 5 µg of shRNA-XT-I using the transfection reagent Exgen 500. Total RNA were extracted 72 h after transfection and expression level of XT-I gene was analyzed by qPCR, as described below. In parallel, 72 h after shRNA-XT-I transfection, cartilage explants were pulsed with ^35^S-sulfate (5 µCi/ml) for the last 6 h prior to GAG analysis [18].

### Cytokine Treatments

Cartilage explants were cultured in six-well plates in DMEM-F12 medium at 37°C in a humidified atmosphere supplemented with 5% CO_2_. After 48 h, explants were serum-starved for 24 h and treated or not (control) with 10 ng/ml of IL-1β or TGF-β1 (Sigma-Aldrich, Saint Quentin, France) for 24 h. At 6 h before the end of the treatment, explants were pulsed with ^35^S-sulfate (10 µCi/ml) prior to GAG analysis [18].

### Cartilage explant culture and measurement of GAG synthesis

Full-depth human articular cartilage slices were washed in serum-free DMEM-F12 medium containing antibiotics, then diced into 1–2-mm^3^ pieces and maintained in a 24-well culture plate (20–30 mg cartilage/well) in 900 µl of DMEM-F12 and 100 µl of PBS, containing 0.1% (w/v) BSA. After 48 h of equilibration, explants were radiolabeled with 10 µCi/ml ^35^S-sulfate (Perkin Elmer, Courtabœuf, France) for 6 h. Explants were then digested in papain and aliquots of ^35^S-labeled digest were precipitated by using cetylpyridinium chloride [18], and the sulfated GAG synthesis rate was measured by liquid scintillation counting.

### Protein extraction and enzymatic activity of XT-I in human cartilage

In brief, cartilage explants were cryosectioned into 10-µm slices using a freezing microtome (Microm HM 440 E) at −20°C. Slices were homogenized using a glass Teflon in cold extraction buffer (500 mM NaCl, 50 mM HEPES, pH 7.2, containing proteinase inhibitor cocktail) at a ratio of 10∶1 (buffer∶tissue) on ice [19]. Protein extraction was carried out at 4°C overnight. The resulting mixture was clarified by centrifugation at 3,000 rpm for 5 min at 4°C. The supernatant was used for measurement of XT-I activity based on the binding of [^14^C]xylose to recombinant bikunin (acceptor substrate), as reported by Weilke et al [20].

### GAG chain analyses by fluorophore-assisted carbohydrate electrophoresis (FACE)

Disaccharide composition of chondroitin sulfate chain was examined on proteinase K-digested cartilage explants by FACE method as described previously [21]. Aliquots containing 5 µg of GAGs, as judged from uronic acid assay [22] from each cartilage sample, were digested with chondroitinase ABC and the disaccharides obtained were fluorotagged and derivatized [21]. Electrophoresis was carried out at 4°C by using miniature vertical slab gels of 29% acrylamide and 1% bisacrylamide at 5 W of constant power per gel. Fluorescent bands were immediately scanned in a UV-light box Gel Doc System (Bio-Rad, Marne la Coquette, France) and quantitated with Bio-Rad QUANTITY ONE software. CS disaccharide standards were from Dextra Laboratories (Reading, U.K.).

### Western blot analysis

Total protein from chondrocytes transfected with pCMV-XT-I or pCMV empty vector was extracted 24 h posttransfection and quantified using Bradford method [23]. Proteins (30 µg/lane) were separated on 12% SDS-PAGE, transferred onto Immobilon® (Millipore, Molsheim, France) membrane and then probed with anti-XT-I antibody (Sigma).

### Statistical analysis

Since OA is a focal disease and because of the reported variations in composition and bioactivity of the cartilage depending on the site studied, the findings of 3 cartilage samples per parameter per joint per patient were averaged and considered as a representative value of that subject [24]. Differences between cartilage samples taken from the lesion, from sites adjacent to the lesion, and from sites distant from the lesion were analyzed by Student's *t*-test (within individual knees) and by Wilcoxon's matched pairs test (between groups) with values of p<0.05 were considered significant.

## Results

### Characterization of lesional and non-lesional areas of human OA cartilage

**Figure 1:**
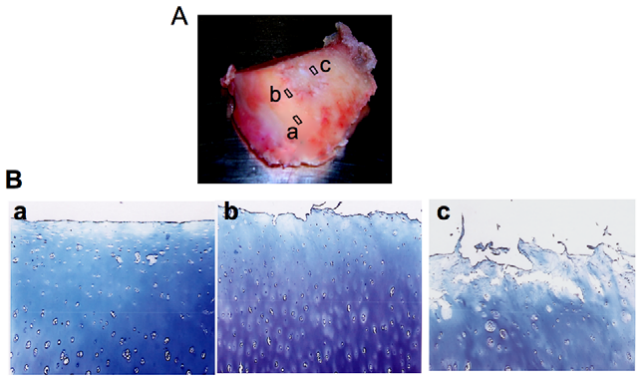
Representative sample of femoral condyle from human OA knee and toluidine blue stained sections. (A) Femoral condyle from human OA knee joint indicating three sites biopsied for further biochemical and molecular analysis; (a) cartilage sample taken from macroscopically unaffected area and distant from the lesion was termed “normal” (b) cartilage sample isolated from areas immediately close to lesion was termed “Next to lesion”; (c) cartilage specimen excised from lesion was termed “late-stage OA”; Cartilage samples were isolated using a 6 mm-biopsy punch. (B) Representative photomicrographs of cartilage sections showing areas from where samples were recovered for toluidine blue staining; (a) cartilage section from a normal area showing a relatively smooth articular surface; (b) section from areas close to lesion showing dense staining for PGs in mid zone of the cartilage where chondrocyte proliferation and activation occurs; (c) cartilage section from lesions showing degraded articular surface, loss of PG staining and chondrocyte cloning (original magnification ×40).

Previous studies of human OA have compared late OA cartilage obtained from joint replacement surgeries to non-OA cartilage from trauma subjects or at autopsy. However, these samples may be collected from subjects that are not corresponding according to age, genetic background or other features, including clinical management of the disease [25]. Also, it is well known that the pattern of disease progression clinically may differ from one patient to another. An alternative method is to evaluate lesional and non-lesional cartilage from neighbouring areas of the same joint [25]–[27]. Three tissue subgroups were objectively segregated according to their degree of degeneration in the same joint. To assess disease severity and cartilage damage part of the cartilage biopsy was processed for histology and graded according to Mankin's scale [15] (surface fibrillation, matrix depletion, cellularity, and the integrity of the tidemark) with a maximum score of 11. No tidemarks were present in our cartilage samples. Cartilage samples taken from degenerative lesions were designated “late disease” (grade 8–11). The sample taken from sites immediately adjacent to lesions was designated “early disease” (grade 3–7) and sample isolated from macroscopically unaffected region and remote from the lesion was termed “normal” (grade 1–2). Cartilage was taken from the same topographical region of the medial condyle and a representative sample of these tissue groups is illustrated in Fig. 1.

### Adaptive increases in sulfated-GAG synthesis in human early OA cartilage

**Figure 2:**
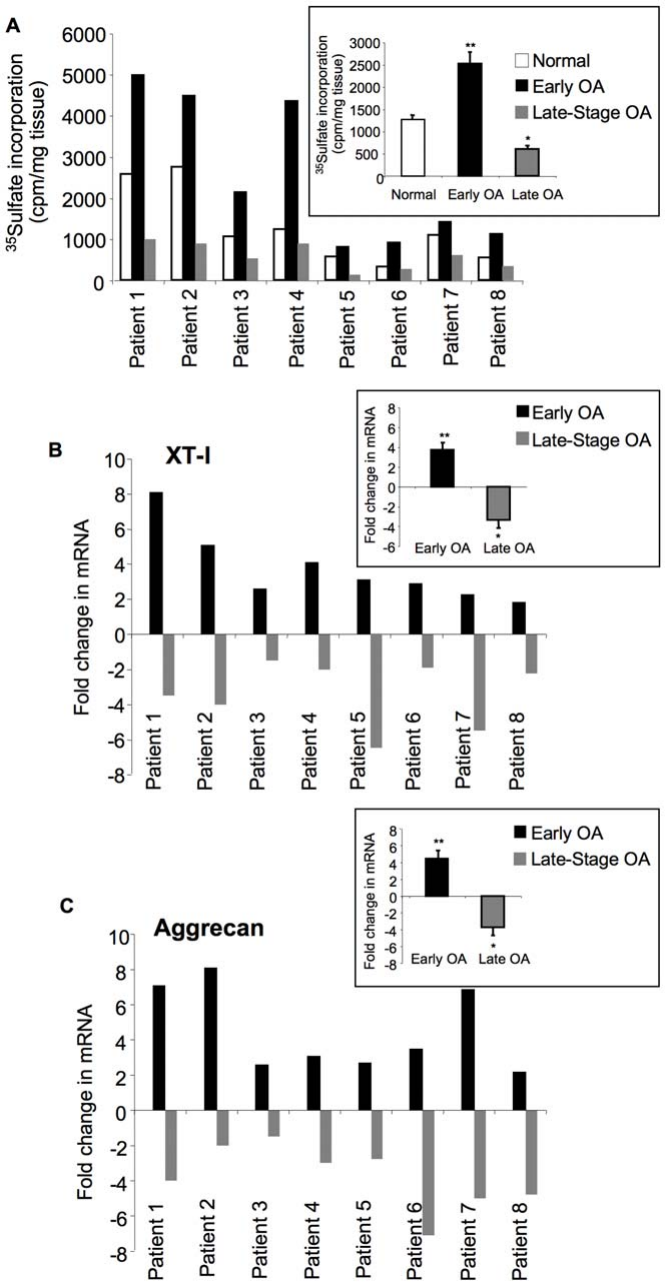
Analysis of GAG synthesis and expression of XT-I and aggrecan genes. (A) GAG synthesis analysis in cartilage from “normal”, early OA and late-stage OA areas for each patient studied by ^35^S-sulfate incorporation. Insert shows the mean of GAG synthesis rate values corresponding to different areas of cartilage sample from all patients. Values are mean ± SEM of 3 experiments per parameter, per joint, per patient. (B) and (C) Analyses of the expression of XT-I and aggrecan genes in cartilage from normal, early OA and late-stage OA areas for each patient. The expression level of the genes was analyzed by qPCR. Measurements were normalized to the control “normal”. Inserts in (B) and (C) show the mean of XT-I and aggrecan gene expression level values corresponding to different areas of cartilage sample from all patients. Values are mean ± SD of 3 experiments per parameter, per joint, per patient. **significantly (*P*<0.01) higher than normal and late groups; §significantly (*P*<0.05) higher than normal and late groups; *significantly (*P*<0.05) lower than normal.

There was a significant increase (1.5- to 3.5-fold) in the rate of sulfated-GAG synthesis in cartilage samples recovered near the site of the lesion (early disease) compared to the samples taken from where normal-appearing cartilage was present for all patients analyzed (Fig. 2*A* and insert), suggesting the hypermetabolic activity next to degenerated tissue. In contrast, our results indicated that the rate of sulfated-GAG synthesis was significantly reduced (1.2- to 3.9-fold) in late OA cartilage (Fig. 2*A* and insert), suggesting that it was less active in terms of sulfated-GAG synthesis. As illustrated in Fig. 1, staining for toluidine blue was also less evident in late OA cartilage section (compare a to b and c). Noteworthy, variations in the rate of GAG anabolism was observed in cartilage from normal areas among the patients studied indicating inter-individual variations in cartilage metabolism (Fig. 2*A*).

### Gene expression patterns of XT-I and aggrecan define the pathologic state of the disease in OA cartilage

To determine if the differences observed in sulfated-GAG synthesis in human OA cartilage were associated with differences in the expression of XT-I responsible for initiation of chondroitin sulfate and heparan sulfate GAG synthesis, we performed qPCR analysis in cartilage samples obtained from areas of “normal”, early disease, and late disease. Depending upon the severity of the lesions, alterations in expression pattern of XT-I gene was observed between diseased and normal areas. Striking increases (1.74- to 8-fold) in the expression level of XT-I gene in OA cartilage were seen in the regions next to lesion compared to normal (Fig. 2*B* and insert). However, in late-stage OA regions, levels of XT-I expression were significantly lower (1.5- to 7-fold) compared to normal (Fig. 2*B* and insert). Thus, XT-I downregulation in diseased cartilage may be one of the major determinants of sulfated GAG depletion and disease progression.

Furthermore, our results also showed that the alterations in aggrecan gene expression paralleled that of XT-I expression with an increase of 2- to 8-fold in regions next to lesion and a decrease of 1.5- to 7-fold in late-stage OA areas (Fig. 2*C* and insert).

### Disease-associated alterations in XT activity in human OA cartilage

**Figure 3:**
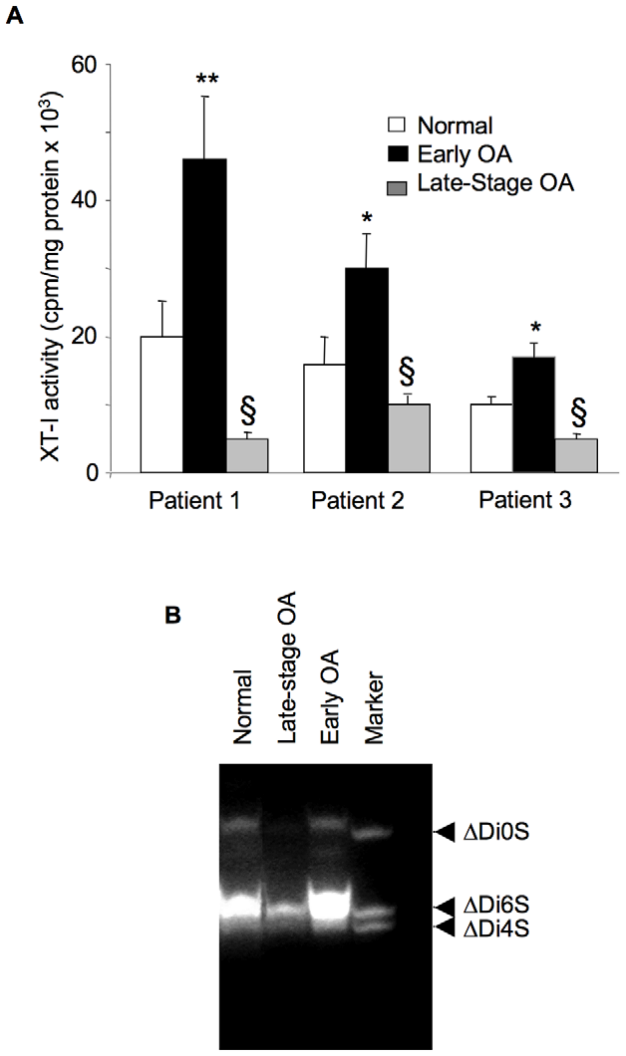
Analysis of xylosyltransferase activity and GAG chain composition. (A) xylosyltransferase activity from normal, early OA and late-stage OA cartilage of three representative patients. Data are mean ± SD of 3 experiments per parameter, per joint, per patient. **Significantly (*P*<0.01) higher than all groups; *significantly (*P*<0.01) higher than normal and late groups; ^§^significantly (*P*<0.05) lower than normal. (B) GAG chain composition in human cartilage explants as determined by FACE. Proteinase K-digested cartilage explants containing equal amounts (5 µg) of GAGs, as determined by uronic acid assay from each group, were digested with chondroitinase ABC and the disaccharides obtained were fluorotagged and derivatized. Disaccharides were run on a monocomposition gel and gels are representative images from 3 experiments per parameter, per joint, per patient. Markers are ΔDi0S, nonsulfated chondroitin-unsaturated disaccharide; ΔDi4S, chondroitin-4-sulfated unsaturated disaccharide; ΔDi6S, chondroitin-6-sulfated unsaturated disaccharide.

As shown in Fig. 3*A*, the xylosyltransferase activity in cartilage from three representative OA patients was increased by 1.2- to 2.5-fold in samples from regions next to lesion when compared with samples from normal regions. In contrast, the activity was decreased by 1.5- to 4-fold in late-stage OA cartilage samples compared with cartilage from normal areas. These results clearly demonstrate that changes in sulfated-GAG synthesis are related to the expression and enzymatic activity of XT-I and to the severity of cartilage lesion.

### FACE analyses of chondroitin sulfate in human OA cartilage

Analysis of the fine structure and disaccharide composition of CS-GAG chains in human cartilage by FACE demonstrated the presence of unsaturated monosulfated (Δdi6S and Δdi4S) and nonsulfated (ΔDi0S) disaccharides from CS/DS chains in normal cartilage. Next, FACE results suggested that 6-sulfated disaccharides were abundant in adult human cartilage. While it is likely that disulfated and higher sulfated products might have been present in the CS-GAG chains, we were unable to profile such units under our assay conditions. Furthermore this study revealed a disease-related quantitative variation of CS disaccharides (Fig. 3*B*). Notably, a strong increase (80%) in disaccharides content occurs in cartilage from regions next to lesion, when compared to normal regions. In contrast, disaccharides content was dramatically reduced (2.5-fold) in tissue obtained from late-stage OA areas (Fig. 3*B*). Thus, it seems very likely that altered gene expression and activity of XT-I might explain the changes observed in CS content in cartilage from areas next to lesion and late-stage OA, suggesting that a functional link may exist between the expression of this glycosyltransferase and sulfated-GAG content in human OA cartilage.

### Down-regulation of XT-I expression impairs sulfated-GAG synthesis in cartilage

**Figure 4:**
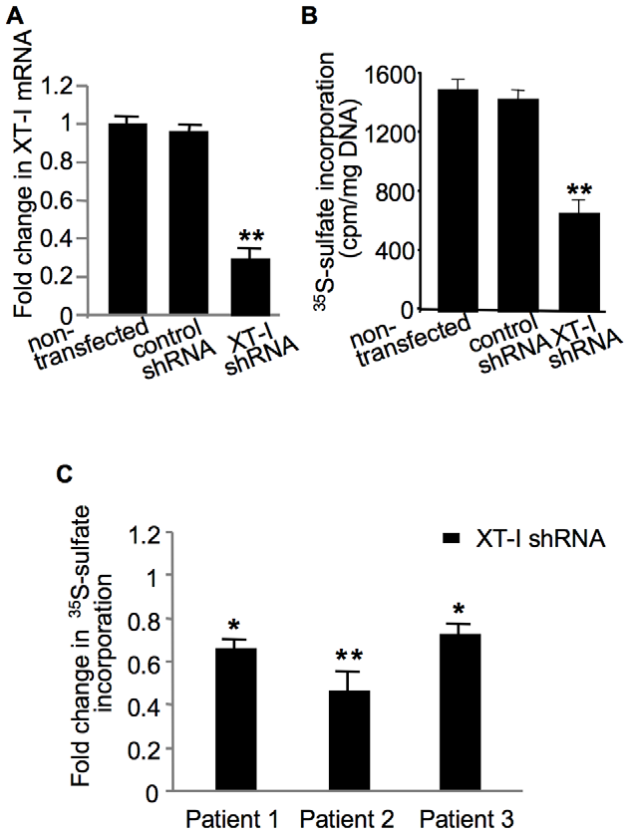
Analysis of the effect of XT-I knockdown on GAG synthesis in chondrocytes and cartilage explants. Cells were transfected with XT-I shRNA, or control shRNA or left untreated and then examined for mRNA expression (A) and GAG synthesis (B). Total RNA was isolated from cells of each group at 72 h posttransfection and XT-I expression level was determined by qPCR analysis. XT-I mRNA levels are represented as a ratio relative to those in negative controls or untreated controls. GAG synthesis was studied by ^35^S-sulfate incorporation. (C) Cartilage explants from normal region of human femoral condyle of three patients were transfected with XT-I shRNA or control shRNA and then GAG synthesis was analyzed by ^35^S-sulfate incorporation. Measurements were normalized to control shRNA. Data represent the mean ± SD of 3 independent observations for each group. **,*Significantly (*P*<0.01, *P*<0.05, respectively) lower than control shRNA.

To determine the importance of XT-I in synthesis of sulfated-GAG in cartilage shRNA was used to transfect chondrocytes and cartilage explants. Quantitative PCR analyses indicated a down-regulation of XT-I mRNA expression of about 70% in shRNA-transfected chondrocytes compared to control groups (Fig. 4*A*). Interestingly, analysis of sulfated-GAG anabolism at 72 h post-transfection showed a reduction of about 50% in shRNA-transfected chondrocytes compared to control groups (Fig. 4*B*) indicating that XT-I plays an important role in sulfated GAG synthesis in chondrocytes. Similarly, transfection of cartilage explants from three different patients with shRNA led to a decrease of sulfated GAG synthesis ranging from 30% to 58% whereas no inhibition was seen in cartilage explants non-transfected or transfected with shRNA control (Fig. 4*C*). Altogether, these results demonstrated the key role of XT-I in sulfated GAG synthesis in human cartilage.

### Gene delivery of XT-I in chondrocytes and cartilage explants stimulates GAG synthesis

**Figure 5:**
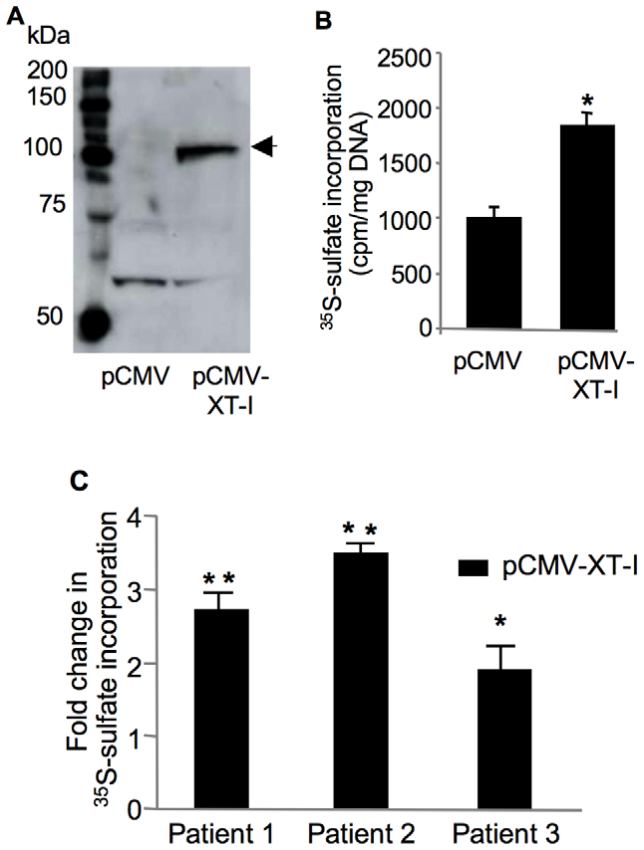
Effect of XT-I overexpression on GAG anabolism in chondrocytes and cartilage. (A) Immunoblot of XT-I protein in pCMV- or pCMV-XT-I-transfected chondrocytes 48 h after transfection. XT-I recombinant protein was indicated by the arrow. (B) GAG synthesis in primary human chondrocytes or (C) in cartilage explants from late OA region of human femoral condyle transfected with pCMV- (control) or pCMV-XT-I vector. XT-I- or mock-transfected cells or cartilage were labeled with ^35^S-sulfate, and the amount of ^35^S-labeled GAGs was measured 48 h posttransfection. Data are mean ± SD of 3 experiments per parameter, per joint, per patient. **,*Significantly (*P*<0.01, *P*<0.05, respectively) higher than controls.

To directly determine the role of XT-I in cartilage GAG anabolism, we overexpressed XT-I in human chondrocytes and cartilage explants. We observed high expression of recombinant XT-I protein in chondrocytes at 48 h after transfection (Fig. 5*A*). Furthermore, analysis of sulfated GAG synthesis showed about 2-fold increase of ^35^S-sulfate incorporation in XT-I-transfected chondrocytes compared to empty vector-transfected cells (Fig. 5*B*) indicating that overexpression of XT-I significantly enhanced sulfated GAG synthesis in chondrocytes (probably by increasing the number of GAG chains that are initiated on a core protein). These results suggested that XT-I enzyme catalyzes a rate-limiting step in sulfated GAG synthesis pathway. Interestingly, transfection of cartilage explants from different patient with pCMV-XT-I vector produced a 2- to 3.5-fold increase in sulfated GAG synthesis compared to control (Fig. 5*C*). These results collectively show that XT-I regulates GAG anabolism in cartilage.

### Expression of IL-1β receptor was down-regulated while TGF-β1 was up-regulated in early OA cartilage

**Figure 6:**
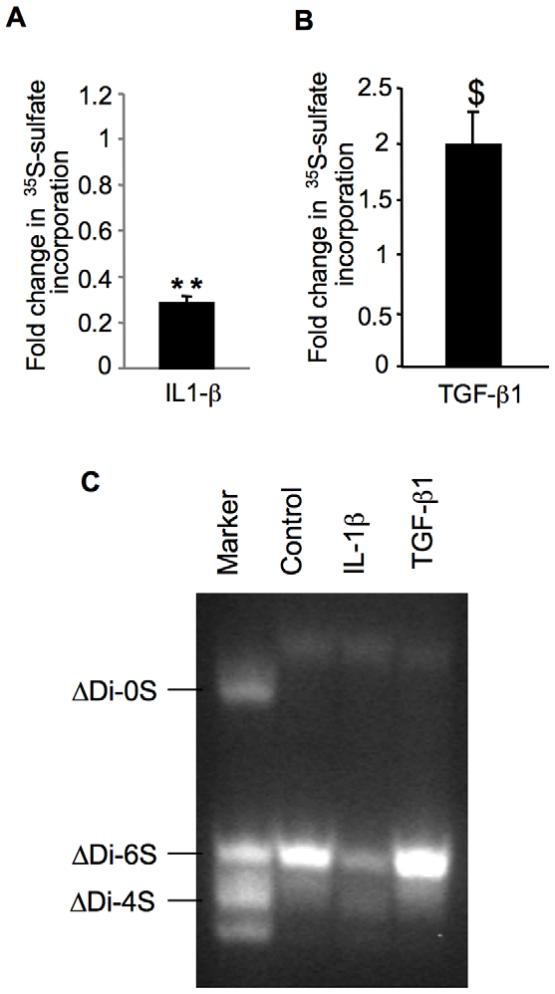
Analysis of the effect of IL-1β and TGF-β1 on GAG synthesis and GAG chain composition. Cartilage explants from normal regions of human femoral condyle were exposed to IL-1β (A) or TGF-β1 (B) for 24 h and then GAG synthesis was measured by ^35^S-sulfate incorporation. Measurements were normalized to control (non treated). Data are mean ± SD of 3 experiments per parameter, per joint, per patient. **Significantly (*P*<0.0018) lower than controls; ^$^significantly (P<0.05) higher than controls. (C) Effect of IL-1β and TGF-β1 on GAG chain composition in human cartilage. Cartilage samples derived from normal regions of human femoral condyle were treated with cytokines for 12 h and analyzed by FACE. Gels are representative images from 3 experiments per parameter, per joint, per patient. Markers are ΔDi0S, nonsulfated chondroitin-unsaturated disaccharide; ΔDi4S, chondroitin-4-sulfated unsaturated disaccharide; ΔDi6S, chondroitin-6-sulfated unsaturated disaccharide.

**Figure 7:**
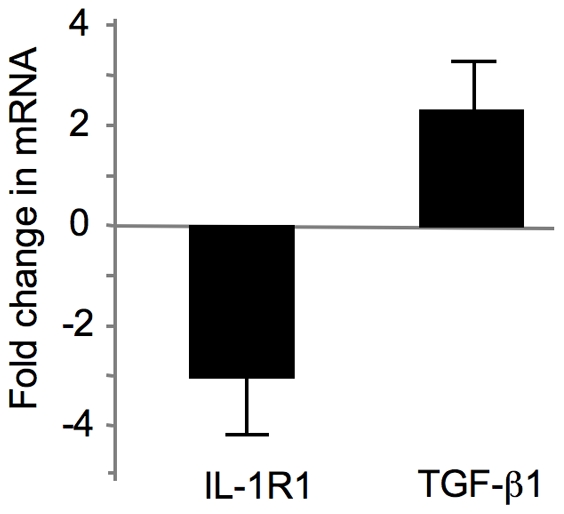
Expression of pro-inflammatory and anti-inflammatory cytokines in human OA cartilage. Total RNA was isolated from human cartilage samples obtained from early OA and late OA regions and the expression of target genes were analyzed by qPCR. Target genes in early OA were compared to late OA and expressed as fold induction. Values are mean ± SD of 3 experiments per parameter per joint per patient.

To determine the link between alteration of XT-I expression and the pathologic state of the disease in OA cartilage potential molecular mechanisms involved were investigated. Proinflammatory cytokine IL-1β and growth factor TGF-β1 regulate GAG anabolism and expression of various factors in chondrocytes. Indeed, treatment of cartilage explants with IL-1β reduced GAG anabolism by about 70% (Fig. 6*A*). In contrast, TGF-β1 treatment led to an increase of GAG synthesis of about 2-fold (Fig. 6*B*). FACE analysis confirmed that TGF-β1 stimulated cartilage CS-GAG synthesis whereas IL-1β treatment inhibited this process (Fig. 6*C*). Interestingly, gene expression analysis indicated significant downregulation of IL-1β receptor (IL-1R1) (3-fold) in early OA cartilage compared to late-stage OA cartilage (Fig. 7). This was associated with an increase of about 2.5-fold in the expression of the anabolic factor TGF-β1, respectively (Fig. 7). These results suggested that cartilage next to lesion not only triggers a mechanism counteracting the effect IL-1β but also stimulates the anabolic activity of chondrocytes by inducing the expression of TGF-β1.

### XT-I gene expression is regulated by IL-1β and TGF-β1 in cartilage

**Figure 8:**
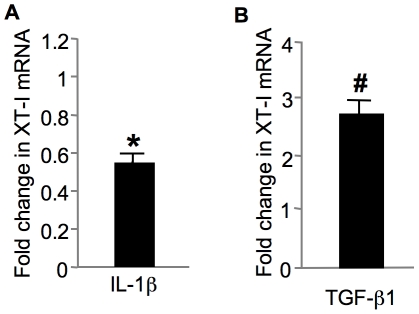
Effect of IL-1β (A) and TGF-β1 (B) on XT-I gene expression in normal articular cartilage. Cartilage samples isolated from normal regions of human femoral condyle was exposed to IL-1β or TGF-β1 and then XT-I gene expression was analyzed by qPCR. Measurements were normalized to control (non treated). Each value represents the mean ± SD of 3 experiments per parameter per joint per patient. *Significantly (*P*<0.05) lower than control group; #significantly (*P*<0.01) higher than control group.

Beside its ability to induce degradation of articular cartilage, the proinflammatory cytokine IL-1β has been shown to suppress the synthesis of PGs by chondrocytes [28], [29]. In contrast the growth factor TGF-β1 is a potent inducer of chondrocyte matrix deposition [30]–[32]. We investigated whether XT-I gene expression is regulated by IL-1β and TGF-β1. Cartilage explants were treated or not with IL-1β and TGF-β1, respectively and XT-I expression was analyzed using quantitative PCR. Our result showed that IL-1β down-regulated the expression of XT-I by about 50% (Fig. 8*A*). In contrast, TGF-β1 induced the expression by about 3-fold (Fig. 8*B*). These results clearly indicated that XT-I gene expression is regulated by the two main cytokines involved in cartilage metabolism.

## Discussion

Our present findings demonstrate that XT-I is likely an important factor in the regulation of susceptibility of humans to cartilage degeneration during OA development and progression. Recently, we showed that loss of XT-I expression increases propensity to loss of sulfated-GAG synthesis and deposition, leading to cartilage destruction in antigen-induced arthritis in rats [13]. Conversely, interruption with XT-I expression disrupts cartilage repair in papain-injected knee joints of rats, providing evidence to date that XT-I plays a key role in replacement of sulfated-GAGs in joint diseases [13]. In order to get more insights, we explore whether XT-I is an important player in regulating sulfated-GAG synthesis and content in human cartilage.

Based on the recognition of wide variation in the degenerative changes in articular cartilages of OA, the use of grading system pioneered by Mankin et al [15] provides a critical assessment of the severity of the OA lesion and the metabolic activity of articular cartilage in OA. There are numerous studies which attempted to correlate biosynthesis changes of aggrecan in OA with pathological findings. The studies reported by Thompson and Oegema [33] reported a decrease in the content of GAG as the histological grade of arthritis increased. In addition studies by Rizkalla et al [34] also reported that in early disease (low grade) there was an increase in GAG synthesis, whereas in late disease it was decreased. Interestingly, using cartilage obtained from different disease areas of femoral condyle from the same OA subject, we describe here, for the first time, the identification of pathology-related alterations in the gene expression and activity of XT-I. The levels of XT-I expression correlated with the severity of degeneration in OA joints. This finding strongly indicated that cartilage close to lesion expressed higher levels of XT-I and increased GAG content, which would explain the high synthesis and content of GAGs in early stages of degeneration. We also showed the association of low expression of the XT-I gene in diseased cartilage where the GAG content was demonstrably reduced. Thus, loss of XT-I expression may set the stage for gradual reduction in sulfated-GAG synthesizing ability of chondrocytes, displaying abnormalities in compensating for the increased degradation of PG-GAGs during OA development. Collectively, our present findings allowed us to extract clinically meaningful information from XT-I gene expression profile and suggest that the pathologic changes involving PG-GAGs in OA may relate to the degree of alteration of XT-I expression and activity.

A striking feature of this study is the upregulation of XT-I gene expression, which becomes significant in view of the present results on human studies indicating that mRNA levels of aggrecan core protein are also increased in early OA. Increased availability of core protein, in the presence of increased XT-I activity, would be expected to increase the transfer of xylose onto core protein acceptor and facilitate the initiation of new CS-GAG chains. This might explain the increase in sulfated-GAG synthesis observations and could offer an explanation for the increase in chondrocyte's capacity to synthesize CS-GAG in early degeneration in OA.

PG-GAG turnover in the cartilage is regulated by a class of proinflammatory and anti-inflammatory cytokines [4], [5]. The proinflammatory cytokine IL-1β was found to induce cartilage loss by reducing PG synthesis [28], [29], [35] and its effects are mediated through a high-affinity cell surface IL-1 receptor type I (IL-1RI) [36]–[38]. Interestingly, our data showed that IL-1RI gene expression was down-regulated in cartilage “next to lesion” compared to late-OA cartilage, indicating a mechanism protecting cartilage against IL-1β-induced effects. In parallel to this protection mechanism, cartilage “next to lesion” produced more TGF-β1, a potent growth factor for cartilage metabolism [39], than late-OA cartilage. Accordingly, previous studies reported that TGF-β1 levels are increased in the first stage of the disease [40]. Although these studies indicated that alterations in PG-GAG anabolism can be influenced by these cytokines, the molecular basis for these diverse actions are poorly understood. Along these lines, our study found that IL-1β may promote cartilage loss by decreasing the expression of XT-I gene with a concomitant reduction in sulfated-GAG synthesis and content in cartilage explants. In this regard, previous studies [41]–[43], including work from our laboratory [13] have shown IL-1β to down-regulate aggrecan core protein, XT-I gene and GAG synthesis in cartilage. In contrast, TGF-β1 treatment results in activation of XT-I gene expression followed by increased sulfated-GAG levels in cartilage explants. Interestingly, regulation of XT-I by TGF-β1 has been also demonstrated in other cell types such as astrocytes [44] and cardiac fibroblasts [45]. It can thus be postulated that the anabolic differences exhibited by IL-1β and TGF-β1 in cartilage explants could originate from their ability to alter XT-I as well as aggrecan core protein expression, with obvious antagonistic relationship to one another in terms of cartilage destruction or repair, respectively. Based on our data, it seems likely that up-regulation of XT-I and sulfated-GAG anabolism in cartilage “next to lesion” was a consequence of combined effects of a protective mechanism against IL-1β and stimulatory effects of TGF-β1.

To unmask the role of XT-I in sulfated-GAG formation and cartilage homeostasis, we studied the effects of targeted suppression of XT-I with gene silencing strategy in chondrocytes and cartilage explants. Our data demonstrates that transfection of chondrocytes with XT-I shRNA reduced their ability to synthesize sulfated-GAGs compared to normal controls. We also provide evidence that cartilage explants in which XT-I expression was silenced by shRNA synthesized lesser amount of sulfated-GAGs compared to non-transfected controls. These results confirm our prediction implicating XT-I as an important enzymatic source of sulfated-GAG production.

From the clinical perspective, development of a targeted therapy with potential cartilage reparative properties is highly significant. Therefore, we wished to determine whether XT-I overexpression represented a valuable therapeutic option to stimulate sulfated-GAG synthesis in OA. We found that forced expression of XT-I strongly upregulated GAG synthesis in chondrocytes and cartilage explants. These in vitro cell culture and cartilage explant data can be used to predict the beneficial effects that XT-I would have on OA chondrocytes. Next, in the hope of achieving clinical benefit in patients with advance OA disease, we determined if we could rescue the low sulfated-GAG synthesis activity of chondrocytes from diseased regions of OA cartilage with over-expression of the XT-I gene. The results demonstrated that forced expression of XT-I significantly improved sulfated-GAG synthesis rate in diseased cartilage. These novel experiments indicate that when XT-I gene is overexpressed in humans with OA these individuals may be capable of presenting increased GAG synthesis activity and provide support in pursuing the demanding goal of cartilage repair.

### Clinical implications and conclusions

Although the patient population examined in this study is not large, nevertheless, the changes in XT-I gene expression we perceive could contribute to many of the observed biochemical characteristics of the disease, particularly for the pathological changes in CS-GAG synthesis and content. Currently, there are no effective therapies to stimulate sulfated-GAG synthesis in cartilage repair, and this deficiency is in part a reflection of a previous lack of understanding of the mechanisms involved in the loss of GAG synthesis in arthritic diseases. Our findings reported here record the protective function of the endogenous XT-I and its role in the maintenance of cartilage GAG homeostasis. Significantly, our results show that the quantitative changes in the expression and activity of XT-I might dictate the disease-related variations in sulfated-GAG synthesis and content. Therefore, pharmacological upregulation of XT-I gene expression along with aggrecan core protein might be expected to increase the amount of functional PG being synthesized and have a therapeutic potential for cartilage repair in OA and also in other degenerative joint diseases.
